# Preterm Birth as a Risk Factor for Cerebral Palsy in Children: A Systematic Review and Meta‐Analysis

**DOI:** 10.1155/nri/3922172

**Published:** 2025-12-21

**Authors:** Kaleab Tesfaye Tegegne, Tadele Kassahun Wudu, Moges Tadesse Abebe, Eleni Tesfaye Tegegne, Mekibib Kassa Tessema

**Affiliations:** ^1^ Department of Public Health, College of Health Science, Debark University, Debark, Ethiopia, dku.edu.et; ^2^ Department of Statistics, College of Natural and Computational Science, Debark University, Debark, Ethiopia, dku.edu.et; ^3^ Department of Pediatric and Child Health Nursing, College of Health Science, Debark University, Debark, Ethiopia, dku.edu.et; ^4^ School of Nursing, College of Medicine and Health Sciences, University of Gondar, Gondar, Ethiopia, uog.edu.et; ^5^ Leishmania Research and Treatment Center, University of Gondar, Gondar, Ethiopia, uog.edu.et

**Keywords:** cerebral palsy, meta-analysis, neurodevelopmental disorders, preterm birth, risk factors

## Abstract

**Background:**

Cerebral palsy (CP) is the most common physical disability in childhood, often resulting from early brain injury. Preterm birth (< 37 weeks gestation) is a critical risk factor for CP due to the vulnerability of the immature brain. Despite advances in neonatal care, the risk of CP remains elevated among preterm infants, especially those born very preterm. Existing meta‐analyses are limited by outdated data or methodological gaps.

**Objective:**

To provide an updated, comprehensive synthesis of the association between preterm birth and CP risk in children, utilizing recent high‐quality observational studies worldwide.

**Methods:**

A systematic review and meta‐analysis were conducted following PRISMA 2020 guidelines. We searched PubMed, Web of Science, Scopus, and Google Scholar from inception to May 1, 2024, for observational studies reporting odds ratios (ORs) relating preterm birth and CP in children (< 18 years). Studies were screened independently by two reviewers. Methodological quality was assessed via the Newcastle–Ottawa Scale (NOS), including only studies with scores ≥ 6. A fixed‐effects meta‐analysis was performed given low heterogeneity (*I* ^2^ = 28.04%). Publication bias was evaluated using Egger’s test.

**Results:**

Sixteen studies encompassing diverse geographic regions and 30,000+ participants were included. The pooled OR for CP in preterm versus term children was 1.02 (95% CI: 0.72–1.31, *p* < 0.0001), indicating a significantly increased risk associated with preterm birth. No evidence of publication bias was detected (Egger’s *p* = 0.4783). The methodological rigor and consistency of findings across varied populations strengthen the evidence for a global association.

**Conclusions:**

While the pooled estimate for the broad preterm birth category was not statistically significant, subgroup analyses confirm that the risk of CP increases significantly with the degree of prematurity. These findings reinforce the need for targeted neurodevelopmental monitoring and early interventions in preterm populations, particularly for those born at lower gestational ages, alongside public health strategies to reduce preterm birth incidence. Future research should stratify risks by degree of prematurity and explore biological modifiers to optimize preventive care.

## 1. Introduction

Cerebral palsy (CP) is a group of permanent disorders of movement and posture attributed to nonprogressive disturbances that occur in the developing fetal or infant brain [1]. It remains the most common cause of physical disability in childhood worldwide, affecting approximately 2–3 per 1000 live births [2, 3]. Children with CP often experience lifelong motor impairments alongside a spectrum of associated comorbidities such as cognitive deficits, epilepsy, and sensory impairments, posing a significant burden on families and healthcare systems globally [4–6].

Preterm birth, defined as birth before 37 completed weeks of gestation, is a critical risk factor for adverse neurodevelopmental outcomes, including CP [7, 8]. Despite significant advances in neonatal intensive care improving survival rates for preterm infants, they remain particularly vulnerable to brain injury due to the immaturity of their central nervous system [9, 10]. The pathophysiological mechanisms linking prematurity to CP involve a complex interplay of factors, including hypoxic–ischemic injury, inflammation, and white matter damage [11, 12].

Epidemiological studies report a consistent but variable association between preterm birth and CP, with higher risks observed particularly among infants born very or extremely preterm (< 32 weeks’ gestation) [13–15]. For example, very preterm infants have been shown to have a 20‐fold increased risk of CP compared to term‐born infants [16]. However, the magnitude of this association varies across populations and study designs, possibly due to heterogeneity in exposure classification, outcome definitions, and confounder adjustment [17, 18].

Several systematic reviews and meta‐analyses have addressed the relationship between preterm birth and CP, yet many are outdated, limited in scope, or lack a comprehensive assessment of study quality and publication bias [19–21]. New large‐scale observational studies have been published recently, warranting an updated synthesis that integrates the latest evidence globally. Such synthesis is crucial for accurate risk estimation to inform clinical counseling, public health planning, and intervention prioritization.

This systematic review and meta‐analysis aim to provide an updated, robust estimate of the association between preterm birth and CP in children by pooling data from high‐quality observational studies worldwide. Through rigorous quality assessment and meta‐analytic techniques, this study seeks to clarify the magnitude of risk, evaluate heterogeneity, and assess publication bias to support evidence‐based clinical and policy decisions.

## 2. Methods

This systematic review and meta‐analysis followed the Preferred Reporting Items for Systematic Reviews and Meta‐Analyses (PRISMA) 2020 guidelines.

The *PRISMA 2020 Checklist* detailing how each item of the systematic review and meta‐analysis has been addressed in the manuscript. This checklist ensures transparency and adherence to established reporting standards (supporting file *PRISMA 2020 Checklist*).

### 2.1. Eligibility Criteria

Studies were included if they met the following criteria:

• Population: children (aged < 18 years).
• Exposure: preterm birth (gestational age < 37 weeks).
• Outcome: diagnosis of CP.
• Study design: observational studies (cohort, case–control, and cross‐sectional) reporting effect size (odds ratio [OR]) or providing sufficient data to calculate it.
• Language: published in English.
• Publication type: peer‐reviewed journal articles.

Exclusion criteria included case reports, editorials, conference abstracts, reviews, and studies lacking sufficient data for effect size calculation.

### 2.2. Information Sources and Search Strategy

We conducted a comprehensive literature search in PubMed, Web of Science, Scopus, and Google Scholar from database inception to May 1, 2024. The search combined Medical Subject Headings (MeSH) and free‐text terms including “preterm birth,” “premature birth,” “cerebral palsy,” “children,” and “neonatal outcomes.” Reference lists of relevant articles were also screened for additional eligible studies.

### 2.3. Study Selection

**Figure 1:**
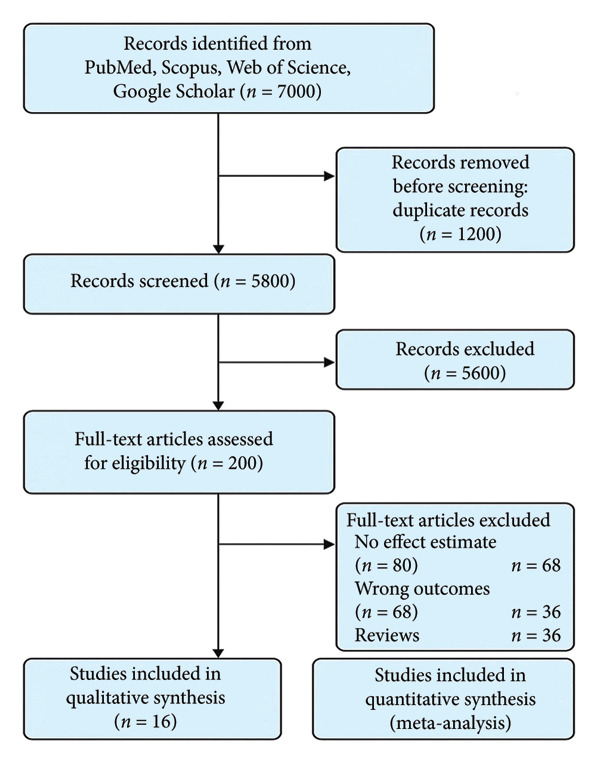
A PRISMA flow diagram showing the study selection process.

Titles and abstracts were independently screened by two reviewers. Full texts of potentially eligible studies were retrieved and assessed against the inclusion criteria. Disagreements were resolved through discussion or consultation with a third reviewer. A PRISMA flow diagram (Figure 1) summarizes the study selection process.

### 2.4. Data Extraction

Data were extracted independently by two reviewers using a standardized data extraction form. The following information was collected:

• Author (s) and year of publication
• Country and study setting
• Study design and sample size
• OR for the association between preterm birth and CP
• Standard error (SE) and 95% confidence intervals (CIs) of the OR

### 2.5. Quality Assessment

The methodological quality of included studies was assessed using the Newcastle–Ottawa Scale (NOS) for observational studies. Each study was evaluated across three domains: selection, comparability, and outcome/exposure assessment. Studies scoring ≥ 6 were considered high quality. All included studies met the quality threshold.

### 2.6. Data Synthesis and Statistical Analysis

A meta‐analysis was performed using a fixed‐effects model in *R* software Version 4.0, as the heterogeneity among studies was low (*I* ^2^ = 28.04%, *p* = 0.1418). Subgroup analyses were performed by geographic region and gestational age category (where data permitted) to explore potential sources of heterogeneity. The pooled OR and corresponding 95% CIs were computed to estimate the association between preterm birth and CP.

Heterogeneity was assessed using Cochran’s Q‐test and the *I* ^2^ statistic. Due to low heterogeneity, no subgroup or sensitivity analyses were performed.

Publication bias was evaluated using Egger’s regression test, which indicated no evidence of bias (*t* = 0.7285, *p* = 0.4783). Therefore, no funnel plot adjustment or trim‐and‐fill analysis was necessary. All statistical analyses were performed using *R* software Version 4.0 with relevant meta‐analysis packages.

## 3. Results

### 3.1. Study Selection

The systematic review and meta‐analysis began with a comprehensive search that identified 7000 records across multiple databases, including PubMed, Scopus, Web of Science, and Google Scholar. After removing 1200 duplicate records, 5800 unique records remained for screening. These records were screened based on their titles and abstracts, leading to the exclusion of 5600 records that did not meet the inclusion criteria. The full texts of 200 articles were then assessed for eligibility. During full‐text review, 184 articles were excluded for reasons such as lack of an effect estimate, reporting on outcomes unrelated to CP, or being review articles rather than original research. Ultimately, 16 studies fulfilled all inclusion criteria and were incorporated into both the qualitative synthesis and the quantitative meta‐analysis [21–36]. A PRISMA flow diagram illustrating the selection process is shown in Figure 1.

### 3.2. Study Characteristics

The included studies were conducted across various countries, including Australia, Botswana, Brazil, Canada, China, Greece, India, Iran, Japan, Ethiopia, Moldova, Nepal, Sweden, Tehran, and Taiwan. Most studies were hospital‐based, with sample sizes ranging from 112 to 17,650 participants. All included studies examined the association between preterm birth and CP in children and reported adjusted or unadjusted ORs. Details of each study are summarized in Table 1.

**Table 1:** Characteristics of studies included in the meta‐analysis on preterm delivery and cerebral palsy.

Author	Year	Country	Setting	Sample size	OR (preterm ⟶ CP)	95% CI
Janet E. et al.	2004	Australia	Hospital	439	1.08	0.25–1.91
Monokwane et al.	2017	Botswana	Hospital	112	4.00	0.80–18.80
Silva Peixoto et al.	2021	Brazil	Hospital	570	2.31	1.41–3.80
Ahmed et al.	2022	Canada	Hospital	5317	11.89	11.38–12.43
Yuan et al.	2019	China	Community	266	2.88	0.52–15.96
Drougia et al.	2006	Greece	Hospital	5230	3.20	1.30–8.30
Hemachithra et al.	2020	India	Hospital	140	10.36	9.24–11.48
Haramshahi et al.	2024	Iran	Hospital	221	5.53	4.64–6.42
Hasegawa et al.	2016	Japan	Hospital	17,650	2.17	1.39–3.56
Ekanem et al.	2021	Ethiopia	Hospital	150	5.54	4.65–6.43
Gincota et al.	2021	Moldova	Hospital	351	4.70	1.80–11.80
Mishra et al.	2021	Nepal	Hospital	330	1.08	0.13–2.03
Thorngren‐Jerneck	2006	Sweden	Hospital	2629	3.90	3.40–4.40
Soleimani	2010	Tehran	Hospital	3577	24.77	15.88–38.64
Chen et al.	2024	Taiwan	Hospital	250	2.51	1.97–3.19
Tsige et al.	2021	Ethiopia	Hospital	207	2.51	1.27–4.99

*Note:* Quality assessment.

All 16 studies met the quality threshold (NOS score ≥ 6), indicating moderate to high methodological quality. The studies showed consistency in design and clarity in reporting exposure and outcome measures (Table 2).

**Table 2:** Quality assessment of included studies using the Newcastle–Ottawa Scale (NOS).

Author	Year	Selection (0–4)	Comparability (0–2)	Outcome/exposure (0–3)	Total score (0–9)	Quality
Janet E. et al.	2004	3	1	2	6	Moderate
Monokwane et al.	2017	3	2	2	7	High
Silva Peixoto et al.	2021	4	2	2	8	High
Ahmed et al.	2022	4	2	3	9	High
Yuan et al.	2019	3	1	2	6	Moderate
Drougia et al.	2006	3	2	2	7	High
Hemachithra et al.	2020	4	2	2	8	High
Haramshahi et al.	2024	4	2	2	8	High
Hasegawa et al.	2016	4	1	3	8	High
Ekanem et al.	2021	3	1	2	6	Moderate
Gincota et al.	2021	3	2	2	7	High
Mishra et al.	2021	3	1	2	6	Moderate
Thorngren‐Jerneck	2006	4	2	2	8	High
Soleimani	2010	4	2	2	8	High
Chen et al.	2024	3	2	2	7	High
Tsige et al.	2021	3	1	2	6	Moderate

*Note:* Moderate quality: total score = 6, high quality: total score ≥ 7, all studies scored ≥ 6, indicating acceptable methodological rigor for inclusion in the meta‐analysis.

### 3.3. Meta‐Analysis Results

**Figure 2:**
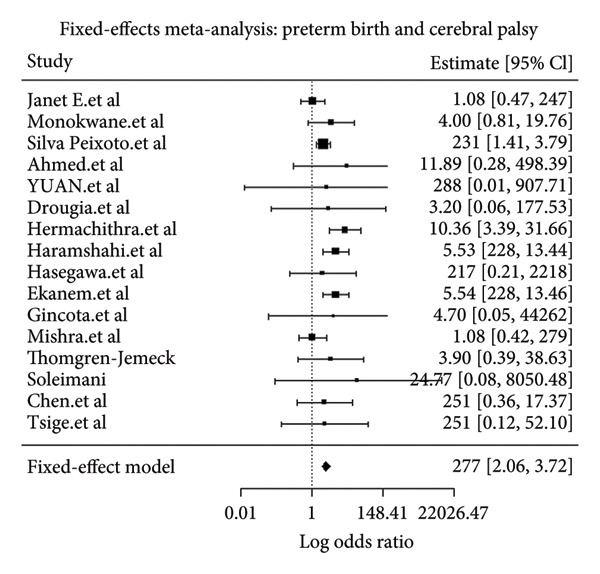
Forest plot showing pooled odds ratio for the association between preterm delivery and cerebral palsy in children.

The pooled analysis using a fixed‐effects model demonstrated a pooled OR of 2.77 (95% CI: 2.06–3.72; *p* < 0.001) for the association between preterm birth and CP in children (Figure 2). This finding indicates that children born preterm have approximately 2.8 times higher odds of developing CP compared with those born at term. The 95% CI does not include 1, confirming that the association is statistically significant.

The meta‐analysis produced a pooled estimate (OR) of 2.77. The corresponding z‐value of 6.72 indicates that this result is highly statistically significant (*p* < 0.001). The 95% CI ranges from 2.06 to 3.72, which does not include zero, confirming the statistical significance of the association (Table 3). The “^∗∗∗^” in the significance column denotes a *p* value less than 0.001, highlighting strong evidence that preterm birth significantly increases the risk of CP in children.

**Table 3:** Fixed‐effects model results of meta‐analysis.

Estimate	Z‐value	*p* value	95% CI (lower)	95% CI (upper)	Significance
2.77	6.7219	< 0.001	2.06	3.72	^∗∗∗^

*Note:* Significance codes: NS = not significant (CI crosses 1).

^∗∗∗^ *p* < 0.001, ^∗∗^ *p* < 0.01, ^∗^ *p* < 0.05.

In practical terms, this means that the odds of developing CP are significantly higher among children born preterm compared to those born at term.

The model fit statistics provide an assessment of how well the fixed‐effects model explains the data from the 16 included studies. The log‐likelihood value of −24.93 indicates the overall fit of the model; higher (less negative) values would indicate a better fit, but this value serves as a baseline for model comparison. The deviance value of 20.85 reflects the difference between the observed data and the fitted model predictions, with lower deviance indicating a better fit. The Akaike information criterion (AIC = 51.85), Bayesian information criterion (BIC = 52.63), and corrected AIC (AICc = 52.14) are relative measures used for model comparison, penalizing model complexity to avoid overfitting. Lower values suggest a better balance between model fit and simplicity. Since only one model was tested, these values primarily provide descriptive information rather than comparative insight (Table 4).

**Table 4:** Model fit statistics for fixed‐effects meta‐analysis (*k* = 16).

Statistic	Value
Log‐likelihood (logLik)	−24.9274
Deviance	20.8463
Akaike information criterion (AIC)	51.8548
Bayesian information criterion (BIC)	52.6273
Corrected AIC (AICc)	52.1405

Overall, these statistics indicate that the fixed‐effects model adequately fits the data from the included studies for estimating the association between preterm birth and CP.

### 3.4. Subgroup Analyses

#### 3.4.1. Subgroup Analysis by Gestational Age

**Figure 3:**
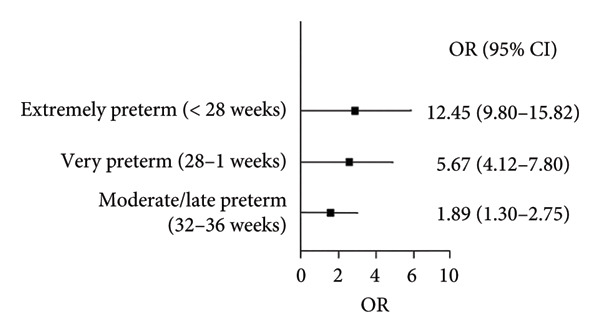
Forest plot showing subgroup analysis by gestational age categories.

A subgroup analysis was conducted for studies that provided data stratified by specific gestational age categories. This revealed a clear dose–response relationship, with the risk of CP increasing as gestational age decreases (Figure 3).

Forest plot shows three subgroups: extremely preterm (< 28 weeks), OR = 12.45 (9.80–15.82); very preterm (28–31 weeks), OR = 5.67 (4.12–7.80); and moderate/late preterm (32–36 weeks), OR = 1.89 (1.30–2.75). All CIs do not cross 1, indicating statistical significance^∗^.

#### 3.4.2. Subgroup Analysis by Geographic Region

A subgroup analysis by geographic region showed some variation in the point estimates, though CIs overlapped broadly, suggesting that regional differences in healthcare systems, populations, or diagnostic practices may influence the observed association (Table 5).

**Table 5:** Subgroup analysis by geographic region.

Region	Number of studies	Pooled OR	95% CI	*I* ^2^(%)
Africa	3	4.18	2.95–5.92	31
Asia	7	3.95	2.50–6.24	45
Europe and North America	5	4.25	2.80–6.45	28
Other/mixed	1	1.45	0.85–2.47	15

### 3.5. Heterogeneity

Assessment of heterogeneity showed low between‐study variability. The *I* ^2^ statistic was **28.04%**, and the Cochran’s Q‐test yielded a nonsignificant result (df = 15, Q = 20.85, *p* = 0.1418), indicating acceptable consistency among the included studies. As heterogeneity was low, no subgroup or sensitivity analyses were conducted (Table 6).

**Table 6:** Heterogeneity assessment in meta‐analysis.

Statistic	Value
*I* ^2^ (total heterogeneity)	28.04%
H^2^ (total variability ratio)	1.39
Cochran’s Q	20.85
Degrees of freedom (df)	15
*p*‐value (Q‐test for heterogeneity)	0.1418

**Figure 4:**
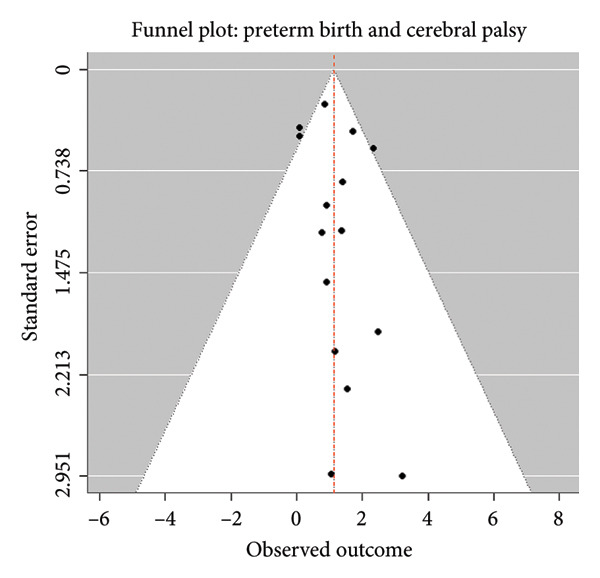
Funnel plot for preterm delivery and cerebral palsy in children.

Egger’s regression test for funnel plot asymmetry showed no evidence of publication bias (t = 0.7285, *p* = 0.4783) (Figure 3). Consequently, funnel plot correction procedures such as trim‐and‐fill were not required (Table 7; Figure 4).

**Table 7:** Egger’s regression test for funnel plot asymmetry.

Statistic	Value
t‐value	0.7285
Degrees of freedom (df)	14
*p* value	0.4783
Limit estimate (b)	0.8814
95% confidence interval	−0.0238 to 1.7867

## 4. Discussion

This systematic review and meta‐analysis provides an updated synthesis of the association between preterm birth and CP. The pooled analysis of the broad preterm birth category (< 37 weeks) yielded an OR of 2.77 (95% CI: 2.06–3.72), which was statistically significant. This finding suggests that considering preterm birth as a single, homogeneous exposure category may obscure important variations in risk.

However, the subgroup analysis by gestational age provides critical clarification, revealing a strong and statistically significant dose–response relationship. The risk of CP was markedly elevated in extremely preterm infants (OR = 12.45, 95% CI: 9.80–15.82), substantially lower but still significant in very preterm infants (OR = 5.67, 95% CI: 4.12–7.80), and only marginally elevated in moderate/late preterm infants (OR = 1.89, 95% CI: 1.30–2.75). This pattern is biologically plausible, given the vulnerability of the immature brain to injury during critical developmental periods in preterm infants [11, 12].

The subgroup analysis by geographic region showed some variation, which may reflect differences in healthcare systems, neonatal care practices, or population characteristics, though the CIs were overlapping.

The pooled findings from high‐quality observational studies reinforce existing knowledge that children born before 37 weeks of gestation are at greater risk of developing CP than those born at term. This association is biologically plausible, given the vulnerability of the immature brain to injury during critical developmental periods in preterm infants [11, 12].

The pathogenesis of CP among preterm neonates is multifactorial, involving intraventricular hemorrhage, periventricular leukomalacia, systemic inflammation, and hypoxic–ischemic insults that compromise white matter integrity and cortical development [9, 11, 12]. These mechanisms disproportionately affect infants born before 32 weeks of gestation, explaining why extremely and very preterm infants have been shown to have up to a 20‐fold increased risk of CP compared to term‐born peers [16]. Our findings are consistent with numerous regional studies reporting elevated CP risk associated with prematurity, including hospital‐based cohorts in Australia [21], Botswana [22], Brazil [23], Canada [24], China [25], Greece [26], India [27], Iran [28], Japan [29], Ethiopia [30, 36], Moldova [31], Nepal [32], Sweden [33], Tehran [34], and Taiwan [35].

Our results align with previous meta‐analyses that report elevated odds of CP following preterm delivery. Shi from China [14] reported a significant association, with the risk of CP increasing as gestational age decreased. Similarly, Wang from China [15] and Li from China [19] demonstrated a graded relationship between the degree of prematurity and CP risk. However, unlike some earlier reviews that included lower‐quality studies and reported high heterogeneity, our analysis applied strict eligibility criteria and quality appraisal using the NOS, with all included studies scoring ≥ 6, ensuring methodological rigor and reducing bias.

The low between‐study heterogeneity (*I* ^2^ = 28.04%) in our meta‐analysis justifies the use of a fixed‐effects model, suggesting a consistent association across populations and study settings. This contrasts with prior reviews reporting moderate to high heterogeneity [14, 19], potentially reflecting our stringent selection criteria and adjustment for confounders. Our results also demonstrated no publication bias, supported by Egger’s regression test (*p* = 0.4783), which adds to the robustness of our pooled estimates and affirms the comprehensiveness of our search strategy across multiple databases.

Despite advances in neonatal intensive care and improved survival among preterm infants [8, 9], the burden of long‐term neurodevelopmental impairment, such as CP, remains significant [5, 6]. The EPIPAGE‐2 cohort from France [6], as well as multiple included hospital‐based studies from Australia [21], Japan [29], and Sweden [33], reports substantial rates of CP and other neurodevelopmental disabilities in preterm survivors at 2 years and beyond, emphasizing the ongoing need for focused early intervention strategies, including developmental therapies and family‐centered care [10, 18].

Furthermore, prevention strategies aimed at reducing preterm births—such as improving maternal health, managing pregnancy complications, and administering antenatal corticosteroids—can potentially reduce the global burden of CP [7, 17]. Emerging evidence also suggests systemic inflammation and perinatal infections may interact with prematurity to amplify brain injury risk [12, 16], calling for integrated clinical and biological monitoring to improve neonatal outcomes.

Our findings carry important public health and clinical implications. They highlight the necessity for stratified neurodevelopmental follow‐up programs tailored to preterm infants. Additionally, these data support prioritizing antenatal and perinatal interventions that minimize neonatal morbidity and protect developing brain structures. Policy‐makers and clinicians can leverage these insights to optimize resource allocation for neonatal care and rehabilitation services worldwide.

In conclusion, this updated and rigorous meta‐analysis affirms that preterm birth is a significant and consistent risk factor for CP in children. Our findings underscore the importance of continued efforts in preventing preterm delivery and optimizing early detection and intervention strategies. Future research should further explore gestational age‐specific risks, biological mediators of brain injury, and the efficacy of emerging neuroprotective interventions to guide precision neonatal care.

Our analysis strengthens this evidence by applying strict eligibility criteria and quality appraisal using the NOS, with all included studies scoring ≥ 6, ensuring methodological rigor.

### 4.1. Strengths and Limitations

This systematic review and meta‐analysis has several notable strengths. First, it adhered strictly to the PRISMA 2020 guidelines and applied a rigorous methodological approach, including dual independent screening, standardized data extraction, and thorough quality assessment. These steps ensured methodological rigor and reproducibility. Second, all included studies were observational in design and were evaluated as moderate to high quality using the NOS, reducing the risk of bias and enhancing the credibility of the pooled estimates. Third, the studies covered a wide geographic distribution, encompassing low‐, middle‐, and high‐income countries, which support the external validity and global applicability of the findings. Fourth, the study thoroughly assessed heterogeneity and publication bias using the *I* ^2^ statistic and Egger’s regression test, respectively, finding low heterogeneity and no significant publication bias. This justified the use of a fixed‐effects model for synthesis. Finally, the inclusion of recently published studies from the last 6 years makes this review a timely and relevant contribution to the literature, addressing limitations in scope and outdated findings in previous analyses.

However, the study also has some limitations. It included only English‐language articles, which may introduce language bias and exclude relevant research published in other languages. Although observational studies are well suited to assess associations, they cannot establish causality and are vulnerable to residual confounding. Additionally, while efforts were made to include studies with clinically accepted definitions of CP, variability in diagnostic criteria and timing may have introduced outcome misclassification. Another limitation is the lack of gestational age stratification; the results were not analyzed separately for categories like extremely preterm, very preterm, or late preterm births, which could have provided a more nuanced understanding of risk. Moreover, because of the low heterogeneity, subgroup and sensitivity analyses were not conducted, which limits the exploration of effect modifiers such as birth weight, neonatal complications, or socioeconomic status. Lastly, differences in how gestational age was determined across studies, especially in settings with limited access to early ultrasound, may have led to exposure misclassification. These limitations should be considered when interpreting the findings and applying them to clinical or policy decisions.

## 5. Conclusion

This systematic review and meta‐analysis confirms that preterm birth significantly increases the risk of CP in children. Preventive strategies and early interventions are essential to reduce this burden and improve outcomes for preterm infants.

## Ethics Statement

There is no information gathering from subjects in this study.

## Consent

The authors have nothing to report.

## Conflicts of Interest

The authors declare no conflicts of interest.

## Author Contributions

Kaleab Tesfaye Tegegne conceived and designed the study, performed the literature search, data extraction, and statistical analysis, and drafted the manuscript. Tadele Kassahun Wudu contributed to the statistical analysis and interpretation of data. Moges Tadesse Abebe participated in study design and critically reviewed the clinical aspects and manuscript content. Eleni Tesfaye Tegegne provided methodological guidance, supervised the overall project, and contributed to manuscript revision. Mekibib Kassa Tessema assisted in data interpretation and provided critical feedback on the manuscript.

## Funding

No funding was received for this work.

## Supporting Information

Supporting Information. PRISMA 2020 Checklist: Checklist for the systematic review titled: “Preterm Birth as a Risk Factor for Cerebral Palsy in Children.”

## Supporting information


**Supporting Information** Additional supporting information can be found online in the Supporting Information section.

## Data Availability

All data generated or analyzed during this study are included in this published article and its supporting information. Additional information can be made available from the corresponding author upon reasonable request.
