# Supplementary material for: Preterm Birth as a Risk Factor for Cerebral Palsy in Children: A Systematic Review and Meta‐Analysis
Source: Neurol Res Int. 2025 Dec 21;2025:3922172. doi: 10.1155/nri/3922172 (PMC12767667; doi:10.1155/nri/3922172)
Supplement: Supplementary file 1 — Supporting Information Additional supporting information can be found online in the Supporting Information section. [file NRI-2025-3922172-s001.docx]

PRISMA 2020 Checklist

Checklist for the systematic review titled: 'Preterm Birth as a Risk Factor for Cerebral Palsy in Children'

Date completed: May 15, 2025

| Section and Topic | Item # | Checklist item | Location where item is reported |
| --- | --- | --- | --- |
| TITLE | 1 | Identify the report as a systematic review. | Preterm Birth as a Risk Factor for Cerebral Palsy in Children – A Systematic Review and Meta-analysis |
| ABSTRACT | 2 | See the PRISMA 2020 for Abstracts checklist. | Structured abstract provided following PRISMA for Abstracts. |
| INTRODUCTION | 3 | Describe the rationale for the review. | Explains the association between preterm birth and cerebral palsy in children. |
| INTRODUCTION | 4 | Provide an explicit statement of the objective(s) or question(s). | To assess whether preterm birth is a significant risk factor for cerebral palsy. |
| METHODS | 5 | Specify inclusion and exclusion criteria and how studies were grouped. | Included studies on children with CP; excluded non-peer-reviewed, animal studies. |
| METHODS | 6 | All information sources (e.g., databases with dates). | PubMed, Scopus, Web of Science, and Google Scholar from January 2000 to December 2023. |
| METHODS | 7 | Search strategy used. | Detailed in Supplementary File 1. |
| METHODS | 8 | Specify the methods used to decide whether a study met inclusion criteria. | Screening by two independent reviewers using pre-defined eligibility criteria. |
| METHODS | 9 | Data collection process. | Standardized data extraction form; two reviewers extracted data independently. |
| METHODS | 10 | List and define all outcomes. | Primary outcome: Odds ratio of CP in preterm vs full-term children. |
| METHODS | 11 | Risk of bias assessment method. | Newcastle-Ottawa Scale used. |
| METHODS | 12 | Effect measures used. | Odds Ratio with 95% Confidence Intervals. |
| METHODS | 13 | Synthesis methods, including statistical methods. | Random-effects meta-analysis using STATA. Heterogeneity assessed with I². |
| RESULTS | 16 | Number of studies included and summary characteristics. | 16 studies included. Characteristics provided in Table 1. |
| RESULTS | 20 | Results of statistical syntheses. | Pooled OR with CI and heterogeneity (I²) reported in forest plot. |
| DISCUSSION | 23 | Summary of evidence. | Preterm birth significantly increases the risk of cerebral palsy in children. |
| DISCUSSION | 25 | Limitations of the evidence and review processes. | Discussed possible publication bias and heterogeneity among included studies. |
| OTHER | 26 | Funding sources. | No funding received. |
| OTHER | 27 | Declaration of competing interests. | Authors declared no competing interests. |
| OTHER | 24 | Registration and protocol. | Protocol registered in PROSPERO (ID: CRD42024567890). |
